# Oxytocin ameliorates cognitive impairments by attenuating excitation/inhibition imbalance of neurotransmitters acting on parvalbumin interneurons in a mouse model of sepsis-associated encephalopathy

**DOI:** 10.7555/JBR.37.20230318

**Published:** 2024-05-29

**Authors:** Renqi Li, Qiuting Zeng, Muhuo Ji, Yue Zhang, Mingjie Mao, Shanwu Feng, Manlin Duan, Zhiqiang Zhou

**Affiliations:** 1 Department of Anesthesiology, Jinling Clinical Medical College of Nanjing Medical University, Nanjing, Jiangsu 210002, China; 2 Department of Anesthesiology, Women's Hospital of Nanjing Medical University, Nanjing Women and Children's Healthcare Hospital, Nanjing, Jiangsu 210004, China; 3 Department of Anesthesiology, Zhongda Hospital, School of Medicine, Southeast University, Nanjing, Jiangsu 210009, China; 4 Department of Anesthesiology, the Second Affiliated Hospital, Nanjing Medical University, Nanjing, Jiangsu 210011, China; 5 Department of Anesthesiology, Jinling Hospital, School of Medicine, Nanjing University, Nanjing, Jiangsu 210002, China

**Keywords:** excitation/inhibition balance, oxytocin, cognitive, sepsis-associated encephalopathy

## Abstract

Inflammation plays a crucial role in the initiation and progression of sepsis and induces alterations in brain neurotransmission, thereby contributing to the development of sepsis-associated encephalopathy (SAE). Parvalbumin (PV) interneurons are pivotal contributors to cognitive processes and have been implicated in various central nervous system dysfunctions, including SAE. Oxytocin, known for its ability to augment the firing rate of gamma-aminobutyric acid (GABA)-ergic interneurons and directly stimulate inhibitory interneurons to enhance the tonic inhibition of pyramidal neurons, has prompted an investigation into its potential therapeutic effects on cognitive dysfunction in SAE. In the current study, we administered intranasal oxytocin to SAE mice induced by lipopolysaccharide. Behavioral assessments, including open field, Y-maze, and fear conditioning, were used to evaluate cognitive performance. Golgi staining revealed hippocampal synaptic deterioration, local field potential recordings showed weakened gamma oscillations, and immunofluorescence staining demonstrated decreased PV expression in the cornu ammonis 1 (CA1) region of the hippocampus following lipopolysaccharide treatment, all of which were alleviated by oxytocin administration. Furthermore, immunofluorescence staining of PV co-localization with vesicular glutamate transporter 1 or vesicular GABA transporter indicated a balanced excitation/inhibition effect of neurotransmitters on PV interneurons after oxytocin administration in the SAE mice, leading to an improved cognitive function. In conclusion, oxytocin treatment improved cognitive function by increasing the number of PV^+^ neurons in the hippocampal CA1 region, restoring the balance of excitatory/inhibitory synaptic transmission on PV interneurons, and enhancing hippocampal CA1 local field potential gamma oscillations. These findings suggest a potential mechanism underlying the beneficial effects of oxytocin in SAE.

## Introduction

Systemic inflammation may induce sepsis-associated encephalopathy (SAE), a common condition among sepsis patients that was first reported in 1827^[1]^. SAE is a prevalent cerebral disorder frequently observed in intensive care units, and its main symptoms are acute, diffuse cognitive dysfunction and altered levels of consciousness level^[2]^. Neuroinflammation, altered neurotransmission, and abnormal cerebral metabolism have been implicated in the pathogenesis of dysfunction and potentially contribute to the development of SAE^[3]^. However, the complex mechanisms underlying the development of SAE remain uncertain and still need to be elucidated.

In the hippocampus, inhibitory parvalbumin (PV) interneurons play a crucial role in facilitating complex cognitive processes, particularly those related to learning and memory. It has been demonstrated that impaired excitation and inhibition of PV interneurons may contribute to cognitive impairments^[4]^. Gamma oscillations are a fundamental and prominent frequency band detected in human electroencephalograms. The synchronous electrical activity of the gamma band originates from the interaction between excitatory pyramidal neurons and inhibitory PV^+^ neurons, accompanied by synchronous inhibitory postsynaptic membrane potentials^[5]^. These gamma oscillations play a crucial role in working memory and are fundamental elements of higher cognitive function and sensory processing^[5–6]^. Synchronization increases during the formation stages of learning and memory^[7]^. For example, Alzheimer's disease interferes with hippocampal gamma oscillation and long-term potentiation, which are recognized as fundamental processes for learning and memory^[8]^. In mouse models of SAE, cognitive impairments have been associated with a decreased expression of PV^[9]^ and alterations in gamma oscillations^[10]^. The decreased gamma oscillations in the cornu ammonis 1 (CA1) region of the hippocampus may be one possible cause of SAE^[4]^. However, the established methodologies that effectively target the excitation of PV^+^ neurons and gamma oscillations to mitigate cognitive dysfunction are currently lacking.

Mounting evidence indicates that oxytocin is involved in the processes of learning and memory^[11]^, including in SAE^[12]^. Oxytocin plays a role in regulating the maturation of excitatory neurons in the hippocampus and maintaining the balance between physiological excitation and inhibition. Disruption of this balance may lead to neurobehavioral disorders^[13–14]^. Oxytocin has a selective stimulatory effect on a specific class of inhibitory interneurons within the CA1 region of the hippocampus^[15]^; it also has an indirect inhibitory regulatory effect on the excitability of pyramidal neurons^[16–17]^. Specifically, oxytocin promotes the synaptic-mediated inhibition of CA1 pyramidal neurons by increasing the firing rate of gamma-aminobutyric acid (GABA)-ergic interneurons located in the pyramidal cell layer^[18]^. Nonetheless, it is unclear whether exposure to oxytocin modulates the balance between excitatory and inhibitory influences on PV^+^ fast-spiking interneurons. It also remains uncertain whether gamma oscillation and cognitive dysfunction can be restored in the SAE mice.

In the current study, we aimed to investigate the therapeutic effects of oxytocin on cognitive dysfunction in SAE mice induced by lipopolysaccharide (LPS). LPS was administered intraperitoneally to induce systemic inflammation, while oxytocin was delivered intranasally. We performed behavioral assessments to evaluate cognitive variations and employed Golgi staining to analyze changes in hippocampal synaptic structure. A linear silicon probe was inserted into the CA1 region of the right hippocampus of each mouse to evaluate gamma oscillatory activity and to record local field potential (LFP). We also quantified the expression of vesicular glutamate transporter 1 (VGluT1) and vesicular GABA transporter (VGAT) on PV^+^ neurons in the CA1 hippocampal region by immunofluorescence (IF) staining, which allowed us to explore the underlying mechanisms of oxytocin's effects on PV interventions and cognitive dysfunction in the SAE mice.

## Materials and methods

### Animals and drug administration

Sixty healthy male C57BL/6 mice (6–8 weeks old) obtained from Gempharmatech Co., Ltd. (Nanjing, Jiangsu, China) were housed in groups (2–4 per cage) at the Animal Center of Jinling Hospital, Nanjing, China. A constant temperature of 22–23 ℃ was maintained under a 12-h light/dark cycle in the cages. *Ad libitum* food and water were available to all mice. Experiments were initiated after a 7-day acclimation period. Male mice were used in the experiments to exclude behavioral effects of estrogen or endogenous oxytocin.

**Figure 1:**
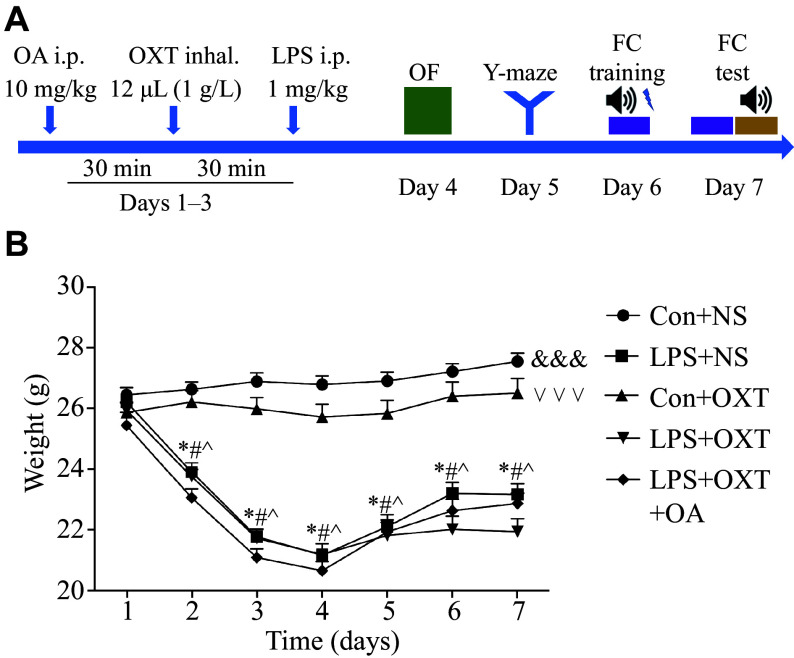
Body weight decreased after LPS administration. A: Schematic timeline of the experimental procedure. B: Body weight fluctuation in each group. Data are presented as mean ± standard deviation (*n* = 12 for each group). Statistical analyses were performed by two-way ANOVA with Bonferroni's test. ^*^P < 0.05 *vs.* day 1 in the LPS + NS group, ^#^*P* < 0.05 *vs.* day 1 in the LPS + OXT group, and ^^^*P* < 0.05 *vs.* day 1 in the LPS + OXT + OA group. Overall differences between groups: ^&&&^*P* < 0.001 between the Con + NS group and the LPS + NS group, ^∨∨∨^*P* < 0.001 between the Con + OXT group and the LPS + OXT group. Abbreviations: OF, open field; FC, fear conditioning; LPS, lipopolysaccharide; OXT, oxytocin; NS, normal saline; OA, oxytocin antagonist.

A concise flow chart of the experimental process is shown in ***Fig. 1A***. Intraperitoneal (i.p.) injections of LPS (1 mg/kg body weight; Cat. #L4130, Sigma-Aldrich, St. Louis, MO, USA) were administered to the mice daily from day 1 to day 3. A total of 12 μL of oxytocin (1 g/L; Cat. #HY-17571A/CS-3775, MedChemExpress, Monmouth Junction, NJ, USA) was administered *via* inhalation into each nasal cavity 30 min before the LPS treatment from day 1 to day 3. A selective oxytocin receptor (OXTR) antagonist (OA) (Cat. #HY-15008, MedChemExpress), dissolved in saline (5 g/L), was administered at a dose of 10 mg/kg body weight 30 min before the oxytocin treatment from day 1 to day 3. The control mice received the same amount of saline.

### Behavioral tests

Every behavioral test was conducted in a darkened and quiet room between 9:00 a.m. and 5:00 p.m. The mice were acclimated to the test chambers 60 min before the test. Experimental grouping was blinded to the investigators during the tests.

#### Open field test

Each mouse was placed in a cube box (XR-XZ301, Shanghai Xinruan Information Technology Co., Ltd., Shanghai, China) to explore freely for 5 min to assess behaviors related to anxiety and motor ability. The open field test was carried out in a 40 cm × 40 cm × 40 cm box. The bottom plate and four walls were all white, and the bottom plate was evenly divided into 4 × 4 small cells horizontally and vertically. The 6th, 7th, 10th, and 11th small cells were defined as the central cells, ordered from the top to the bottom and the left to the right. The test was conducted in a quiet environment (the experimenter observed from a distance, and there was no movement or sound during the test). The light level in the box was set to a low level (approximately 30 lux). After being placed gently in the center of the box, each mouse was free to explore for 5 min, and the movement of each mouse was recorded and analyzed during the test. After each test, the inner wall and bottom of the box were wiped with alcohol gauze to ensure that the experimental results of the next mouse were not affected by residual information (size, urine, odor, *etc.*).

#### Y-maze test

Each mouse was placed in the Y-maze (XR-XY1032, Shanghai Xinruan Information Technology Co., Ltd.) to explore freely for 8 min to evaluate motor ability and working memory. The Y-maze was constructed in a 35 cm × 5 cm × 15 cm (length × width × height) apparatus. The maze consisted of three identical arms (A, B, and C), and the angle between each pair of arms was 120°. On day 4, to evaluate spatial working memory, each mouse was placed at the far end of the same arm and allowed to explore freely for 8 min. The arm sequence of arm entries was recorded. A thorough cleaning of the apparatus was conducted after each trial using 75% ethanol. It was called the spontaneous alternation performance if the mouse entered at least three different arms. The spontaneous alternation percentage was calculated following the formula: (number of alternating triads)/(total number of arm entries − 2) × 100%.

#### Fear conditioning test

To assess the hippocampal-dependent memory, each mouse was placed in the chamber (XR-XC404, Shanghai Xinruan Information Technology Co., Ltd.) for 5 min without any tone or foot shock to test their contextual fear conditioning. On day 5, the mice were individually placed in a chamber measuring 30 cm × 30 cm × 45 cm (length × width × height) for training. On day 6, the mice were tested to assess their associative memory. During training, each mouse was allowed to explore freely for 3 min. This was followed by a 30 s tone (70 dB, 3000 Hz), and then a 2 s foot shock (0.75 mA). After the conditioning test, each mouse was given an additional 30 s of free movement before being returned to its home cage. At 24 h after training, the mice were placed back in the chamber for 5 min without any tone or foot shock to test their contextual fear conditioning. Two hours after the contextual memory test, the mice were placed in a new chamber with different shapes and odors to assess their cued fear memory. After 3 min of acclimating to the new environment, the mice were exposed to a 180 s tone (70 dB, 3000 Hz) without any accompanying foot shock. Freezing time, defined as the absence of movement except for respiration, was recorded during all tests. The chamber was thoroughly cleaned using 75% ethanol at the end of each trial.

#### LFP recording

A linear silicon probe was planted in the CA1 region of the right hippocampus of each mouse, and then LFP was used to obtain the gamma band power. After anesthetization with an i.p. administration of pentobarbital sodium (40 mg/kg body weight), an apparatus with left and right ear rods was used to fix each mouse. The bregma and posterior fontanelle were exposed after the cranial crest was cut along the longitudinal incision of each mouse. A 16-channel linear silicon probe was planted in the CA1 region of the right hippocampus after craniotomy and removal of the dura mater. According to the brain atlas of the mouse, stereotaxic coordinates were determined (posterior, 2.2 mm; lateral, 1.5 mm; and depth, 1.6 mm). During the indicated time points, the LFPs of mice were recorded in home cages with the OmniPlex recording system (Hong Kong Plexon Co., Ltd., Hong Kong, China). After being filtered at 0.3–300 Hz, 2 kHz was selected for amplifying and digitizing the LFP signals. The powerline artifact was removed using a 50-Hz notching filter. The wideband recordings were down-sampled at 1000 Hz for LFP analysis. In the current study, gamma oscillations were filtered as follows: slow gamma (30–50 Hz), medium gamma (50–80 Hz), and fast gamma (80–140 Hz). The power spectral density (PSD) was derived from the wave. Data analysis was conducted with Neuroexplorer software (Plexon Inc., Dallas, TX, USA).

### IF staining

In the CA1 hippocampal region, the expression of PV^+^ neurons, the co-expression of OXTR with PV^+^ or CaMKⅡ^+^ neurons, and the co-expression of PV^+^ neurons with vesicular glutamate transporter 1 (VGluT1) or vesicular GABA transporter (VGAT) were detected by IF staining. Following behavioral tests on day 7, the mice were deeply anesthetized and then perfused transcardially with cold saline followed by paraformaldehyde in phosphate-buffered saline. After harvesting, the brain tissues were processed with a series of ethanol and xylene washes for dehydration and clearing. The tissue blocks were then immersed in paraffin and rolled in the groove to ensure that all sides of the tissue blocks were uniformly contacted with the paraffin liquid. All tissues were preserved for slicing, sectioned into 8-μm-thick slices, and carefully placed on glass slides. The slides underwent a series of treatments, including immersions in xylene and ethanol, and antigen retrieval in EDTA buffer. During the above process, the drying of slices because of the excessive evaporation of buffer solution was avoided. After thorough washing, the sections were circled and sequentially incubated with primary and secondary antibodies before DAPI staining and mounting. All slides were visualized with a microscope (Scope A1, ZEISS, Germany).

To ensure the electrode was placed in the hippocampus CA1 area with the method described above, the probe was dipped in the red fluorescence probe solution (Dil, 10 μmol/L; Cat. #C1036, Beyotime, Shanghai, China) before being implanted in the brain. The electrode was extracted gently after implantation was completed, and the hippocampus was sliced for IF staining to check if the electrode position was accurate.

The following primary antibodies were used: mouse monoclonal anti-VGluT1 (1∶1000 dilution; Cat. #135011, Synaptic Systems, Goettingen, Germany), mouse monoclonal anti-VGAT (1∶150 dilution; Cat. #SC-393373, Santa Cruz Biotechnology, Dallas, Texas, USA), rabbit polyclonal anti-PV (1∶400 dilution; Cat. #26521-1-AP, Proteintech, Wuhan, Hubei, China), mouse monoclonal anti-PV (1∶500 dilution; Cat. #MAB1572, Millipore, Billerica, MA, USA), rabbit polyclonal anti-PV (1∶1000 dilution; Cat. #ab11427, Abcam, Cambridge, UK), rabbit monoclonal anti-CaMKⅡ (1∶400 dilution; Cat. #ab134041, Abcam), and rabbit polyclonal anti-oxytocin R (1∶500 dilution; Cat. #bs-1314R, Bioss, Beijing, China). The following secondary antibodies were used: Alexa Fluor 488-conjugated donkey anti-rabbit IgG (H+L) (1∶600 dilution; Cat. #711-545-152, Jackson ImmuneResearch, PA, USA), CoraLite488-conjugated goat anti-mouse IgG (H+L) (1∶800 dilution; Cat. #SA00013-1, Proteintech), CoraLite594-conjugated goat anti-mouse IgG (H+L) (1∶500 dilution; Cat. #SA00013-3, Proteintech), and CoraLite594-conjugated goat anti-rabbit IgG (H+L) (1∶500 dilution; Cat. #SA00013-4, Proteintech).

### Golgi staining

Golgi staining was performed to test the hippocampal synaptic structure changes in each group. A Golgi Stain Kit (#PK401, FD Neuro Technologies, Columbia, MD, USA) was used for Golgi staining. The experiment was conducted following the manufacturer's instructions. The neurons and dendritic spines were captured with the microscope (Scope A1, ZEISS) under 200× and oil-immersion 1000× magnification. The slides from the hippocampus CA1 area in each group were chosen for scanning and photographing. Two slices with a clear background and uniform staining from each animal and three clear visual fields of the hippocampal CA1 area in each slice were randomly selected. Image J software and the plug-in Sholl analysis were used to simulate the dendritic morphology of the cell. Concentric circles were drawn with the cell body taken as the center, and 10 μm of length was perceived as the increment amplitude of radius, until the end of the longest dendrite. The complexity of dendrites was analyzed through the intersections of dendritic branches and each concentric circle. The length of the dendritic branches of the corresponding segments (longer than 10 μm) was measured, and the density of dendritic spines was calculated.

### Statistical analysis

Data were presented as mean ± standard deviation. Statistical analysis was performed using SPSS (edition 25; IBM Corp., Armonk, NY, USA). The mean values between groups were compared by repeated-measurement ANOVA or two-way ANOVA. For pairwise comparisons among multiple groups, the Bonferroni method was used. Statistical significance was indicated by *P* < 0.05.

## Results

### Body weight fluctuated after the LPS challenge

First, we investigated the effects of LPS on the mortality and weight of the mice. No animal died in any group. As shown in ***Fig. 1B***, body weight fluctuated after the LPS challenge. In comparison to day 1, the body weight in the LPS + normal saline (NS) group, LPS + oxytocin (OXT) group, and LPS + OXT + OA group was significantly lower on days 2 to 7 (all *P-*values < 0.05). Overall significant differences (all *P-*values < 0.001) were observed between the Con + NS group and the LPS + NS group, between the Con + NS group and the LPS + OXT group, between the Con + NS group and the LPS + OXT + OA group, as well as between the Con + OXT group and the LPS + OXT group.

### Oxytocin ameliorated cognitive performance in the LPS-challenged mice

#### Motor ability and anxiety-like behavior were not impaired in each group of mice

**Figure 2:**
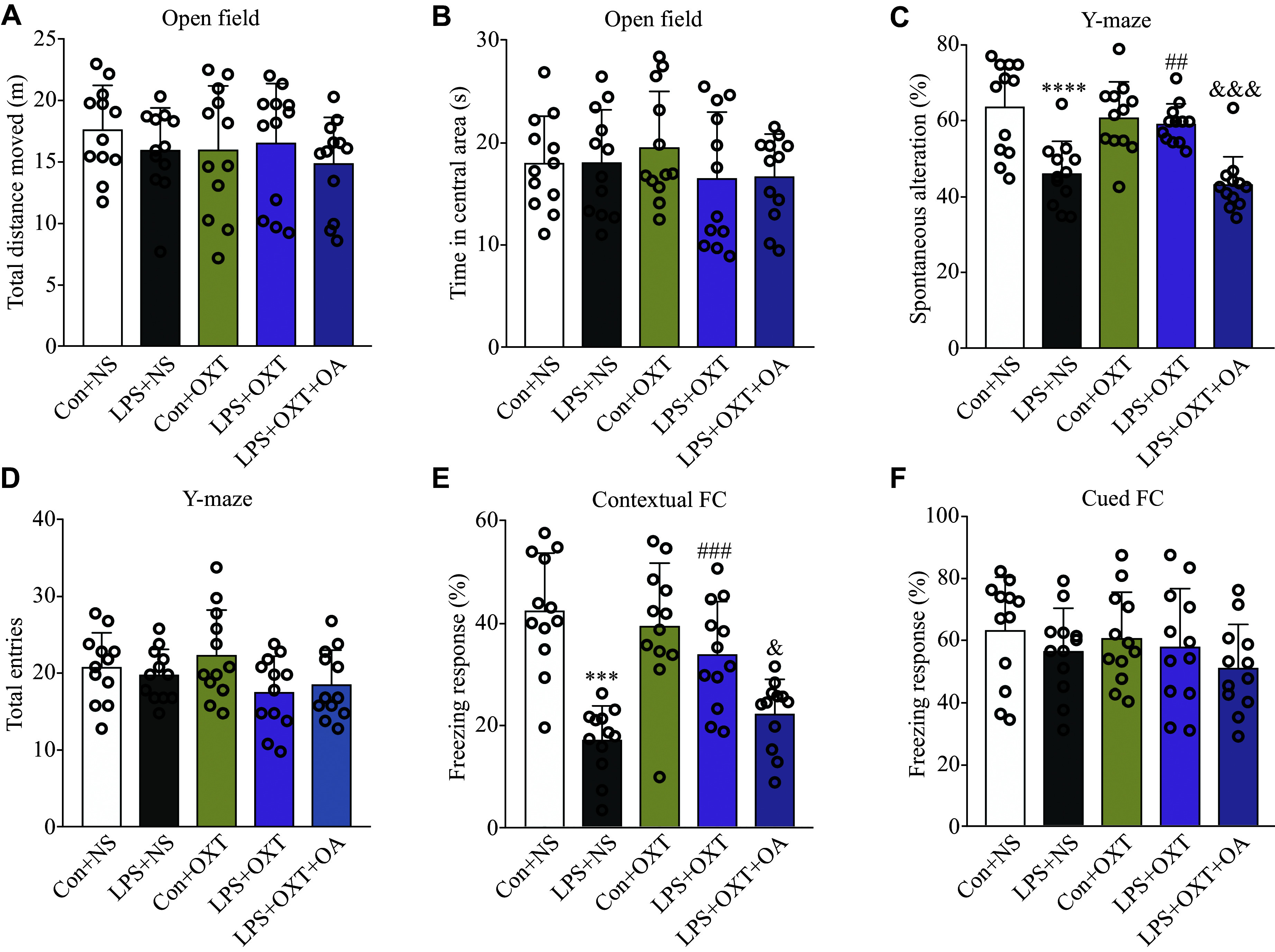
Oxytocin ameliorated cognitive performance declines in the SAE mice. A and B: Motor ability and anxiety-like behavior in open field tests. C: The spontaneous alteration percentage of spatial working memory based on Y-maze tests. D: Total entries in Y-maze tests. E: Hippocampus-dependent memory performance based on contextual fear conditioning tests. The freezing response percentage was observed among the different groups of mice. F: Non-hippocampus-dependent memory performance based on cued fear conditioning tests. Data are presented as mean ± standard deviation. Statistical analyses were performed by two-way ANOVA with Bonferroni's test. ^ ***^P < 0.001 and ^****^P < 0.0001 *vs.* the Con + NS group; ^##^*P* < 0.01 and ^###^*P* < 0.001 *vs.* the LPS + NS group; ^&^*P* < 0.05 and ^&&&^*P* < 0.001 *vs.* the LPS + OXT group. *n* = 12 for each group. Abbreviations: SAE, sepsis-associated encephalopathy; Con, control; LPS, lipopolysaccharide; OXT, oxytocin; NS, normal saline; OA, oxytocin antagonist.

No significant differences were observed in the total distance moved (*P* = 0.61) or time spent in the central area (*P* = 0.62) among the groups during the open field test (***Fig. 2A*** and ***2B***). The results indicate that LPS challenge as well as all the other treatments do not affect the motor ability or anxiety-like behavior of the mice.

#### Oxytocin ameliorated spatial working memory impaired in the SAE mice

The spontaneous alteration percentage was significantly lower (*P* < 0.0001) in the LPS + NS group than in the Con + NS group, while intranasal inhalation of oxytocin significantly reversed this decrease (*P* = 0.0063) in the LPS + OXT group (***Fig. 2C***). However, OA administration blocked the effect of oxytocin, resulting in a significantly decreased spontaneous alteration percentage (*P* = 0.0005) in the LPS + OXT + OA group, compared with the LPS + OXT group (***Fig. 2C***). No significant difference (*P* = 0.11) was found in the total entries among the groups in the Y-maze test (***Fig. 2D***). These results indicate that oxytocin may alleviate spatial working memory impairment in the LPS-induced SAE mice.

#### Oxytocin ameliorated hippocampal-dependent memory impairment in the SAE mice

In contextual fear conditioning tests, the freezing response percentage of mice was significantly lower (*P* < 0.0001) in the LPS + NS group, compared with the Con + NS group, while intranasal inhalation of oxytocin significantly reversed this decrease(*P* = 0.0009) in the LPS + OXT group (***Fig. 2E***). However, OA administration blocked the effect of oxytocin, resulting in a significantly lower freezing response percentage (*P* = 0.0398) in the LPS + OXT + OA group, compared with the LPS + OXT group. These results indicate that oxytocin may alleviate the hippocampal-dependent memory impairment in the LPS-induced SAE mice.

#### Non-hippocampal dependent memory was not impaired in any group of the mice

In cued fear conditioning tests, we observed no significant difference in the freezing response percentage among the groups (*P* = 0.4041; ***Fig. 2F***). This result indicates that the LPS challenge as well as all the other treatments does not impair the non-hippocampal dependent memory in the mice.

### Oxytocin ameliorated hippocampal synaptic structure deterioration in the SAE mice

**Figure 3:**
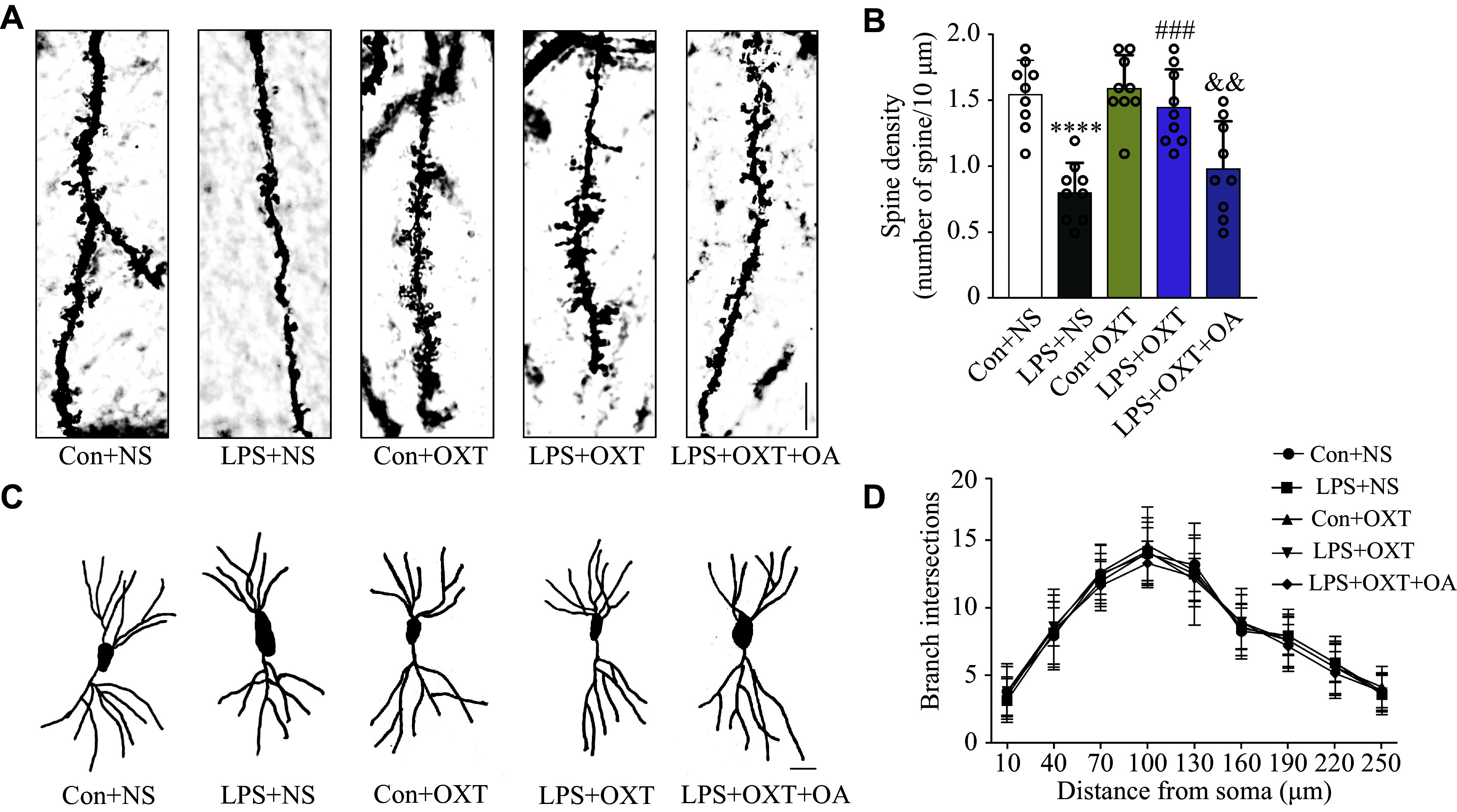
Oxytocin ameliorated hippocampal synaptic structure deterioration in the SAE mice. A: Representative images of the dendritic spine of CA1 neurons in different groups of mice. Scale bar, 10 μm. B: Bar charts represent the number of dendritic spines per 10 μm length of primary dendrites in the CA1 hippocampal region of mice in different groups. C: Representative dendritic complexity of the two-dimensional distribution of neuronal dendrites in the CA1 hippocampal region among different groups of mice. Scale bar, 20 μm. D: Branch intersections of hippocampal CA1 neurons in different groups of mice. Data are shown as mean ± standard deviation (*n* = 9 for each group). Statistical analyses were performed by two-way ANOVA with Bonferroni's test. ^****^P < 0.0001 *vs.* the Con + NS group; ^###^*P* < 0.001 *vs.* the LPS + NS group; ^&&^*P* < 0.01 *vs.* the LPS + OXT group. Abbreviations: CA1, cornu ammonis 1; SAE, sepsis-associated encephalopathy; Con, control; LPS, lipopolysaccharide; OXT, oxytocin; NS, normal saline; OA, oxytocin antagonist.

The results of Golgi staining showed a significant decrease in the density of the dendritic spines in the LPS + NS group, compared with the Con + NS group (*P* < 0.0001), while intranasal inhalation of oxytocin significantly reversed this reduction (*P* = 0.0001) in the LPS + OXT group (***Fig. 3A*** and ***3B***). However, OA administration blocked the effect of oxytocin, resulting in a significantly reduced density of the dendritic spines (*P* = 0.0083) in the LPS + OXT + OA group, compared with the LPS + OXT group (***Fig. 3A*** and ***3B***). No significant differences (*P* > 0.05) in dendritic complexity were observed among the groups (***Fig. 3C*** and ***3D***). The results indicate that oxytocin may alleviate the LPS-induced alterations in hippocampal synaptic structure in the LPS-induced SAE mice.

### Oxytocin increased the slow and medium gamma band power in the CA1 hippocampal region in the SAE mice

**Figure 4:**
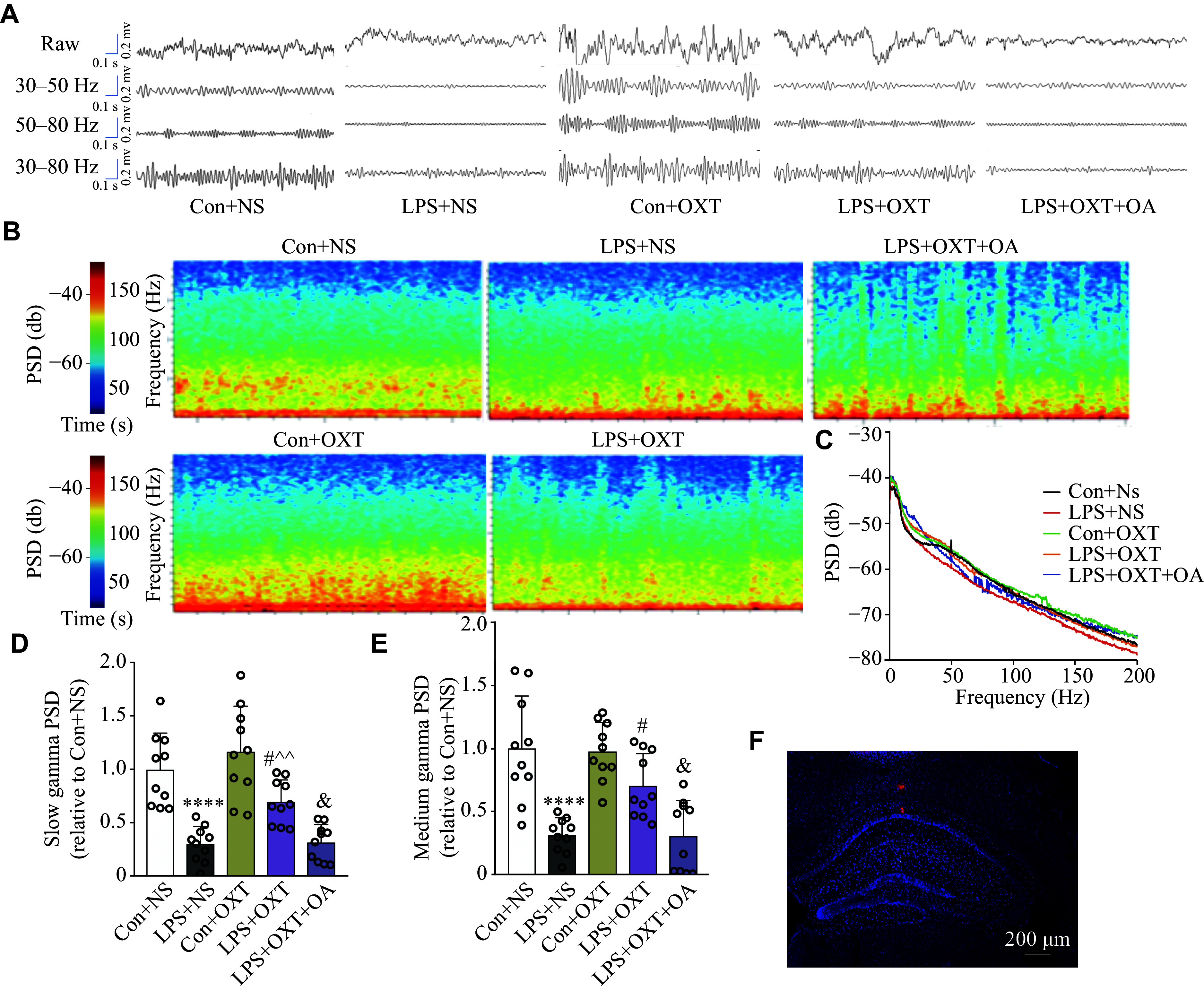
Oxytocin increased the slow gamma band power in the CA1 hippocampal region of the SAE mice. A: Representative original and different frequencies of filtered waveforms of LFP in the CA1 hippocampal region. B: The example PSD of LFP heat map in the CA1 hippocampal region among the groups. C: The PSD of LFP in the CA1 hippocampal region among the groups. D: The slow gamma PSD (30–50 Hz) of LFP in the CA1 hippocampal region among groups. E: The medium gamma PSD (50–80 Hz) of LFP in the CA1 hippocampal region among the groups. F: Representative red fluorescence of electrodes' notch in the CA1 hippocampal region. Scale bar, 200 μm. Data are shown as mean ± standard deviation (*n* = 10). Statistical analyses were performed by two-way ANOVA with Bonferroni's test. ^****^P < 0.0001 *vs.* the Con + NS group; ^#^*P* < 0.05 *vs.* the LPS + NS group; ^^^^*P* < 0.01 *vs.* the Con + OXT group; and ^ &^*P* < 0.05 *vs.* the LPS + OXT group. Abbreviations: CA1, cornu ammonis 1; SAE, sepsis-associated encephalopathy; LFP, local field potential; PSD, power spectral density; Con, control; LPS, lipopolysaccharide; OXT, oxytocin; NS, normal saline; OA, oxytocin antagonist.

LFP was used to assess the gamma band power. Gamma band waves at different frequencies were filtered from original LFP in the CA1 hippocampal region (***Fig. 4A***). The example PSD of LFP heat map (***Fig. 4B***) and the PSDs of LFP in the CA1 hippocampal region (***Fig. 4C***) among the groups were derived. The PSD of the slow gamma band in the LPS + NS group was significantly decreased (*P* < 0.0001), compared with the Con + NS group, while intranasal inhalation of oxytocin significantly reversed this reduction (*P* = 0.0314) in the LPS + OXT group, although it remained lower (*P* = 0.0062) than that in the Con + NS group (***Fig. 4D***). OA administration blocked the effect of oxytocin and significantly decreased the PSD of the slow gamma band (*P* = 0.0426) in the LPS + OXT + OA group, compared with the LPS + OXT group (***Fig. 4D***). Additionally, the PSD of the medium gamma band in the LPS + NS group was also significantly decreased (*P* < 0.0001), compared with the Con + NS group, while intranasal inhalation of oxytocin significantly reversed this reduction (*P* = 0.0304) in the LPS + OXT group (***Fig. 4E***). OA administration blocked the effect of oxytocin and significantly decreased the PSD of the medium gamma band (*P* = 0.0264) in the LPS + OXT + OA group, compared with the LPS + OXT group (***Fig. 4E***). After extracting the probe, the hippocampus was sliced and analyzed by fluorescence microscopy. The red fluorescence (Dil) demonstrated the electrode's precise positioning (***Fig. 4F***). The results indicate that a decreased power of slow and medium gamma bands in the CA1 hippocampal region in the SAE mice may be ameliorated by oxytocin.

### Oxytocin increased the PV^+^ neuron intensity in the CA1 hippocampal region of the SAE mice

**Figure 5:**
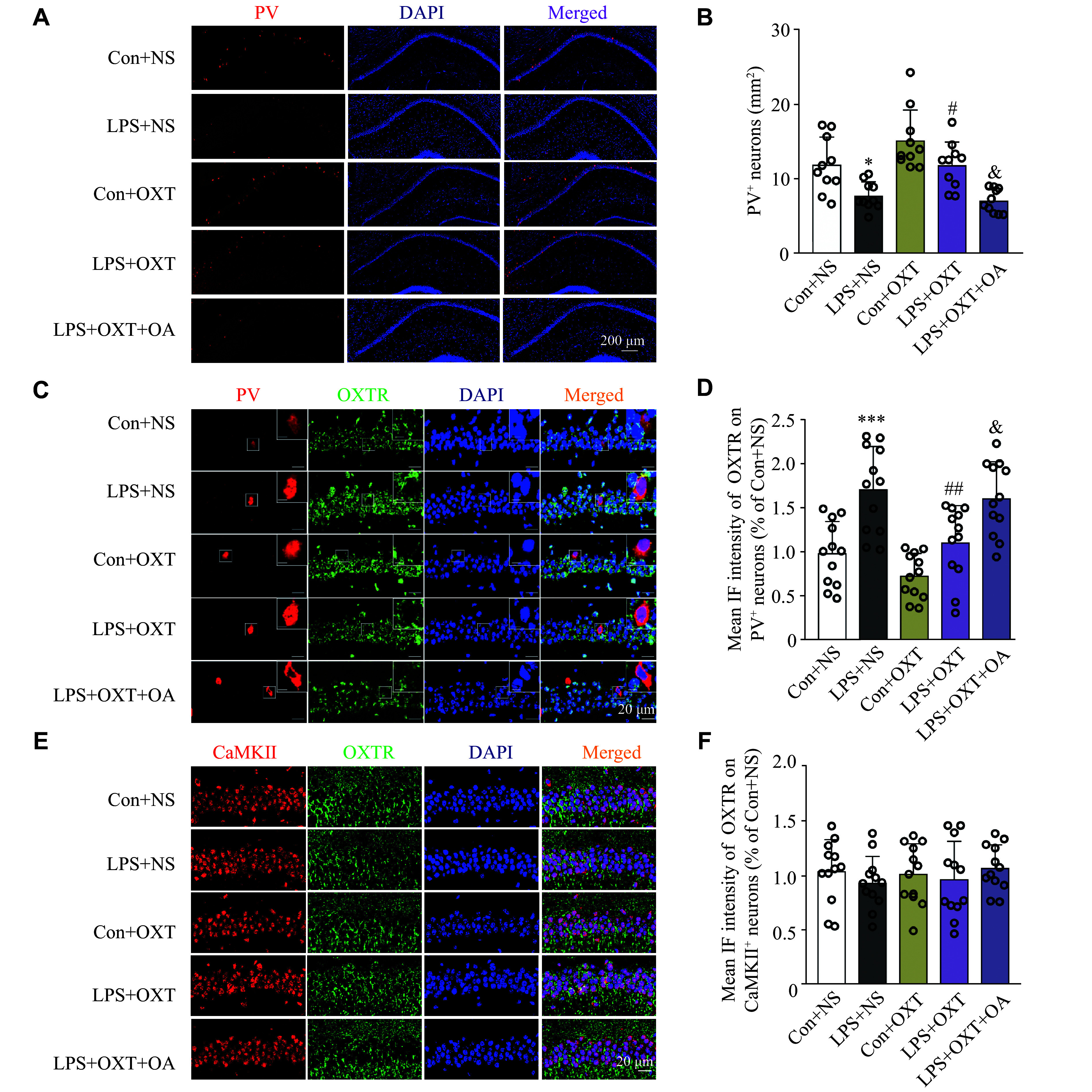
Intranasal inhalation of oxytocin increased the PV^+^ neuron intensity and decreased the expression of OXTR on PV^+^ neurons in the CA1 hippocampal region of SAE mice. A: Representative IF images of PV^+^ neurons in the CA1 hippocampal region. Scale bar, 200 μm. B: PV^+^ neuron density in the CA1 hippocampal region among the groups of mice. Data are shown as mean ± standard deviation (*n* = 10 for each group). Statistical analyses were performed by two-way ANOVA with Bonferroni's test. ^*^P < 0.05 *vs.* the Con + NS group; ^#^*P* < 0.05 *vs.* the LPS + NS group; ^&^*P* < 0.05 *vs.* the LPS + OXT group. C: Representative IF images of OXTR on PV^+^ neurons in the CA1 hippocampal region. Scale bar, 20 μm. The magnified images are in the upper right area. Scale bar, 5 μm. D: Mean IF intensity of OXTR on PV^+^ neurons in the CA1 hippocampal region among the groups of mice. Data are shown as mean ± standard deviation (*n* = 12). Statistical analyses were performed by two-way ANOVA with Bonferroni's test. ^***^P < 0.001 *vs.* the Con + NS group; ^##^*P* < 0.01 *vs.* the LPS + NS group; ^&^*P* < 0.05 *vs.* the LPS + OXT group. Scale bar, 20 μm. The magnified images are in the upper right area. Scale bar, 5 μm. E: Representative IF images of OXTR on CaMKⅡ^+^ neurons in the CA1 hippocampal region. Scale bar, 20 μm. F: Mean IF intensity of OXTR on CaMKⅡ^+^ neurons in the CA1 hippocampal region among the groups of mice. Data are shown as mean ± standard deviation (*n* = 12 for each group). Statistical analyses were performed by two-way ANOVA with Bonferroni's test. Abbreviations: CA1, cornu ammonis 1; PV, parvalbumin; LPS, lipopolysaccharide; OXT, oxytocin; NS, normal saline; OA, oxytocin antagonist; SAE, sepsis-associated encephalopathy; OXTR, oxytocin receptor; CaMKⅡ, calcium/calmodulin-dependent protein kinase Ⅱ; IF, immunofluorescence.

As shown in ***Fig. 5A*** and ***5B***, the density of PV^+^ cells in the CA1 hippocampal region was significantly decreased (*P* = 0.0394) in the LPS + NS group, compared with the Con + NS group, while intranasal inhalation of oxytocin significantly reversed this reduction (*P* = 0.0443) in the LPS + OXT group. The OA administration blocked the effect of oxytocin and significantly decreased (*P* = 0.0111) the PV^+^ neuron density in the CA1 hippocampal region in the LPS + OXT + OA group, compared with the LPS + OXT group. These results indicate that a decrease in the quantity of PV^+^ neurons in the CA1 hippocampal region in the LPS-induced SAE mice may be ameliorated by oxytocin.

### Oxytocin decreased the expression of OXTR on PV^+^ neurons in the CA1 hippocampal region of the SAE mice

As shown in ***Fig. 5C*** and ***5D***, the expression of OXTR on PV^+^ neurons in the CA1 hippocampal region was significantly increased (*P* = 0.0004) in the LPS + NS group, compared with the Con + NS group, while intranasal inhalation of oxytocin significantly reversed this increase (*P* = 0.0044) in the LPS + OXT group. The OA administration blocked the effect of oxytocin and significantly increased (*P* = 0.0304) the expression of OXTR on PV^+^ neurons in the CA1 hippocampal region in the LPS + OXT + OA group, compared with the LPS + OXT group. The results indicate that an increase in OXTR quantity on PV^+^ neurons in the CA1 hippocampal region of LPS-induced SAE mice may be reversed by oxytocin.

### Oxytocin did not change the expression of OXTR on CaMKⅡ^+^ neurons in the CA1 hippocampal region of the SAE mice

As shown in ***Fig. 5E*** and ***5F***, no significant differences (*P* > 0.05) were observed among the groups of mice. This indicates that the LPS challenge as well as all the other treatments do not impair the expression of OXTR on the excitatory neurons in the CA1 hippocampal region of the mice.

### Oxytocin inhalation attenuated the imbalance of the excitatory VGluT1 and inhibitory VGAT on PV^+^ neurons in the CA1 hippocampal region of the SAE mice

**Figure 6:**
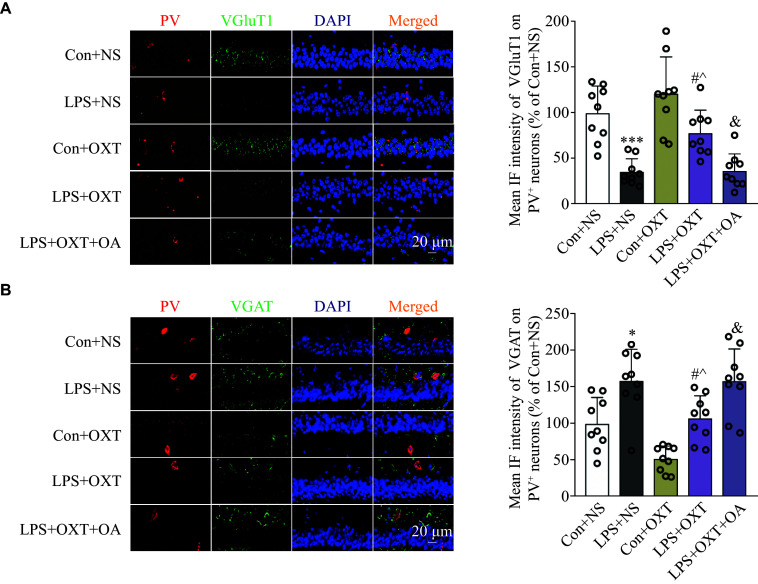
Immunofluorescence-based co-localizations of PV and VGluT1 or VGAT in the CA1 hippocampal region. A: The co-localization of PV and VGluT1 in the CA1 hippocampal region among the groups of mice. B: The co-localization of PV and VGAT in the CA1 hippocampal region among the groups of mice. Data are shown as mean ± standard deviation (*n* = 9 for each group). Statistical analyses were performed by two-way ANOVA with Bonferroni's test. ^*^P < 0.05 and ^***^P < 0.001 *vs.* the Con + NS group; ^#^*P* < 0.05 *vs.* the LPS + NS group; ^^^*P* < 0.05 *vs.* the Con + OXT group; and ^&^*P* < 0.05 *vs.* the LPS + OXT group. Scale bar, 20 μm. Abbreviations: CA1, cornu ammonis 1; PV, parvalbumin; VGluT1, vesicular glutamate transporter-1; VGAT, vesicular GABA transporter; Con, control; LPS, lipopolysaccharide; OXT, oxytocin; NS, normal saline; OA, oxytocin antagonist.

As shown in ***Fig. 6***, compared with the Con + NS group, the co-localization of PV with VGluT1 was decreased significantly (*P* = 0.0001) in the LPS + NS group, whereas the co-localization of PV and VGAT was increased significantly (*P* = 0.0127). Compared with the LPS + NS group, the co-localization of PV and VGluT1 was increased significantly (*P* = 0.0204) in oxytocin-rescued mice from the LPS + OXT group, while the co-localization of PV and VGAT was decreased significantly (*P* = 0.0428). Compared with the Con + OXT group, the co-localization of PV and VGluT1 was decreased significantly (*P* = 0.0185) in the LPS + OXT group, while the co-localization of PV and VGAT was increased significantly (*P* = 0.0213). Compared with the LPS + OXT group, the co-localization of PV and VGluT1 was decreased significantly (*P* = 0.0251) in the LPS + OXT + OA group, while the co-localization of PV and VGAT was increased significantly (*P* = 0.0454). These results indicate that oxytocin may attenuate the imbalance of the excitatory VGluT1 and inhibitory VGAT on PV^+^ neurons in the CA1 hippocampal region in the LPS-induced SAE mice.

## Discussion

In the current study, we established an SAE model by intraperitoneal injection of LPS and investigated the therapeutic effect of intranasal administration of oxytocin on SAE and its potential mechanisms. We observed that post-sepsis oxytocin inhalation alleviated cognitive dysfunction and behavioral changes in cognitive function in the SAE mice. The changes in LFP and gamma oscillations in the hippocampal CA1 area of the mice were involved in this process. Furthermore, alterations in the excitatory and inhibitory neurotransmitters acting on PV interneurons may be the underlying mechanisms. Therefore, oxytocin inhalation provides a new perspective for targeted interventions in SAE.

Systemic inflammation induces encephalopathy, and its underlying mechanisms are multi-faceted and complex^[3]^. Intraperitoneal injection of LPS is a commonly used method for establishing SAE models. In our study, a single intraperitoneal injection of LPS at a dosage of 1 mg/kg for three consecutive days caused systemic inflammation and damage to the blood-brain barrier in mice, ultimately resulting in SAE^[19]^. Behavioral tests confirmed the successful establishment of a stable and reliable SAE animal model. The current study aimed to investigate the LPS-induced encephalopathy in the hippocampal CA1 region, which plays a pivotal role in cognitive function modulation through its connections from the dentate gyrus (DG) to the CA3 and subsequently to the CA1^[20–23]^. Furthermore, the CA1 region is known to be more vulnerable to oxidative stress, apoptosis, and ischemia, compared with the DG and CA3 regions^[24–27]^.

Intranasally delivered oxytocin may quickly reach the brain and take effect, as it bypasses the first-pass metabolism and the blood-brain barrier. Compared with intraperitoneal injection, intranasal oxytocin administration significantly increased oxytocin concentration in the dorsal hippocampus within the first 30 min, maintaining elevated levels for several hours^[28]^. In addition, oxytocin has also been reported to act on the hippocampus to improve cognitive dysfunction^[11–12]^. Furthermore, oxytocin regulates the development of excitatory neurons in the hippocampus and contributes to maintaining a physiological excitatory/inhibitory balance, the disruption of which may lead to neurobehavioral disorders^[13]^. In the current study, we demonstrated that oxytocin treatment upregulated PV expression and enhanced gamma oscillations, thereby improving cognitive function in SAE mice.

In recent years, numerous studies have indicated an association between the diminished PV expression levels and disrupted gamma oscillation patterns with cognitive impairments^[29–30]^. Normal cerebral function relies on the dynamic balance between excitatory and inhibitory neurons. The synchronized electrical activity of excitatory and inhibitory neurons forms the basis of gamma-band synchronous electrical activity^[5]^, which is crucial for spatial working memory^[31]^. Gamma rhythms also recruit neurons and glial responses to mitigate pathological changes associated with Alzheimer's disease^[6,32]^ and improve cognition^[33]^. Fast-spiking PV interneurons are a type of inhibitory GABA-ergic neuron and their synchronous electrical activities are important for maintaining the excitation/inhibition dynamic balance; moreover, the fast-spiking PV interneurons are correlated with gamma oscillations^[8,34]^. In the current study, we observed alterations in gamma oscillations of LFPs in the hippocampal CA1 region, prompting us to further investigate the function of gamma-related PV^+^ neurons and the mechanisms underlying their altered excitability in SAE.

Importantly, glutamate acts as an excitatory neurotransmitter, while GABA acts as an inhibitory neurotransmitter within the central nervous system^[35–36]^. In the current study, we chose and quantified the presynaptic markers of both excitatory and inhibitory neurons, namely VGluT1 and VGAT. VGluT1 is located in the vesicles of the excitatory synapses, while VGAT is located in the vesicles of the inhibitory synapses^[37]^. These transporters significantly contribute to the regulation of excitation/inhibition neurotransmission by influencing vesicle filling and quantum size^[38]^. Based on the quantified levels, it can be inferred that imbalanced bidirectional effects on PV interneurons are derived from the enhanced inhibitory VGAT neurotransmissions and the declined excitatory VGLUT1 neurotransmissions in the LPS-challenged mice. These effects may mediate the excitation/inhibition imbalance of PV interneurons at the synaptic level. In contrast, oxytocin alleviated excitation/inhibition imbalance by enhancing the VGluT1 effects on PV, while decreasing the VGAT effects.

However, the current study also has some limitations. Firstly, it has been reported that LPS administration causes splenomegaly and elevates levels of pro-inflammatory cytokines in the blood, with positive correlations between spleen weight and cytokine levels in the blood^[39–40]^. However, it was not measured whether oxytocin attenuated splenomegaly or systemic inflammation in the LPS-treated mice in the current study. Mice mortality did not occur in any group in the current study. However, the mortality may reach 70% in SAE patients in intensive care units^[3]^. In the current study, cerebral dysfunction was derived from a relatively low level of inflammation, and it was easy to investigate the effect of oxytocin on cerebral function without dealing with other confounding factors. Secondly, the balance of excitation and inhibition in the brain is controlled by a variety of neurons, and the inhibitory PV interneurons are GABA-ergic neurons that account for 40%–50% of all GABA-ergic neurons in the brain. Although inhibitory interneurons account for a small proportion of total brain neurons, most of which are excitatory pyramidal neurons, the neural circuits are in a state of balance between synaptic excitation and inhibition; this balance is usually based on fast glutamate and slow GABA inputs, and inhibition is two to six times stronger than excitation^[41]^. Gamma oscillation is a basic form of synchronous electrical activity in the nerve micro-loop, which plays an important role in maintaining cognitive function. PV interneurons have the characteristics of fast discharge and are the main force producing gamma oscillation^[42]^. It is impossible to study the effects of all excitatory and inhibitory neurons at one time. Therefore, we have chosen to study the gamma oscillation that plays a significant role in neural function. In gamma oscillations, the change in slope at the specific slow gamma frequency corresponds to the alterations in excitation/inhibition balance^[43]^. Therefore, in the current study, the frequency band of slow gamma oscillations was selected to investigate the PSD change in the LPS-challenged mice. Finally, OXTR is expressed in several interneurons, such as somatostatin (SST), PV, and pyramidal neurons^[44]^, and some interneurons, such as SST cells, may be specifically activated by OXTR^[45]^. Furthermore, SST neurons inhibit PV neurons and also receive excitation inputs from pyramidal cells^[46–47]^. In the current study, only PV interneurons and the related gamma oscillations were investigated, and it remains unknown whether other interneurons activated by OXTR are involved in the process of SAE. We found that the expression of PV decreased, and that the co-localization of VGluT1 and VGAT on PV^+^ neurons decreased, indicating changes in neurotransmitters acting on the postsynaptic membrane. However, it remains unclear whether the synapses are decreased. Consequently, it needs to be demonstrated by measuring PSD95, GAD67, and Gephyrin.

It has been reported that OXTR is distributed in both the cerebral cortex and hippocampus^[48]^. In the central nervous system, oxytocin inhibited inflammation of microglial cells and attenuated the activation of microglial cells in the LPS-treated mice^[49]^. Consistent with a recent study, the LPS-challenged mice showed higher levels of OXTR on PV^+^ neurons in the CA1 hippocampal region in the current study^[12]^. The increased OXTR expression in the brain may play a role in cerebral anti-inflammation. However, further studies are needed to elucidate the mechanisms underlying the activation or inhibition of VGluT1 and VGAT on PV^+^ neurons.

**Figure 7:**
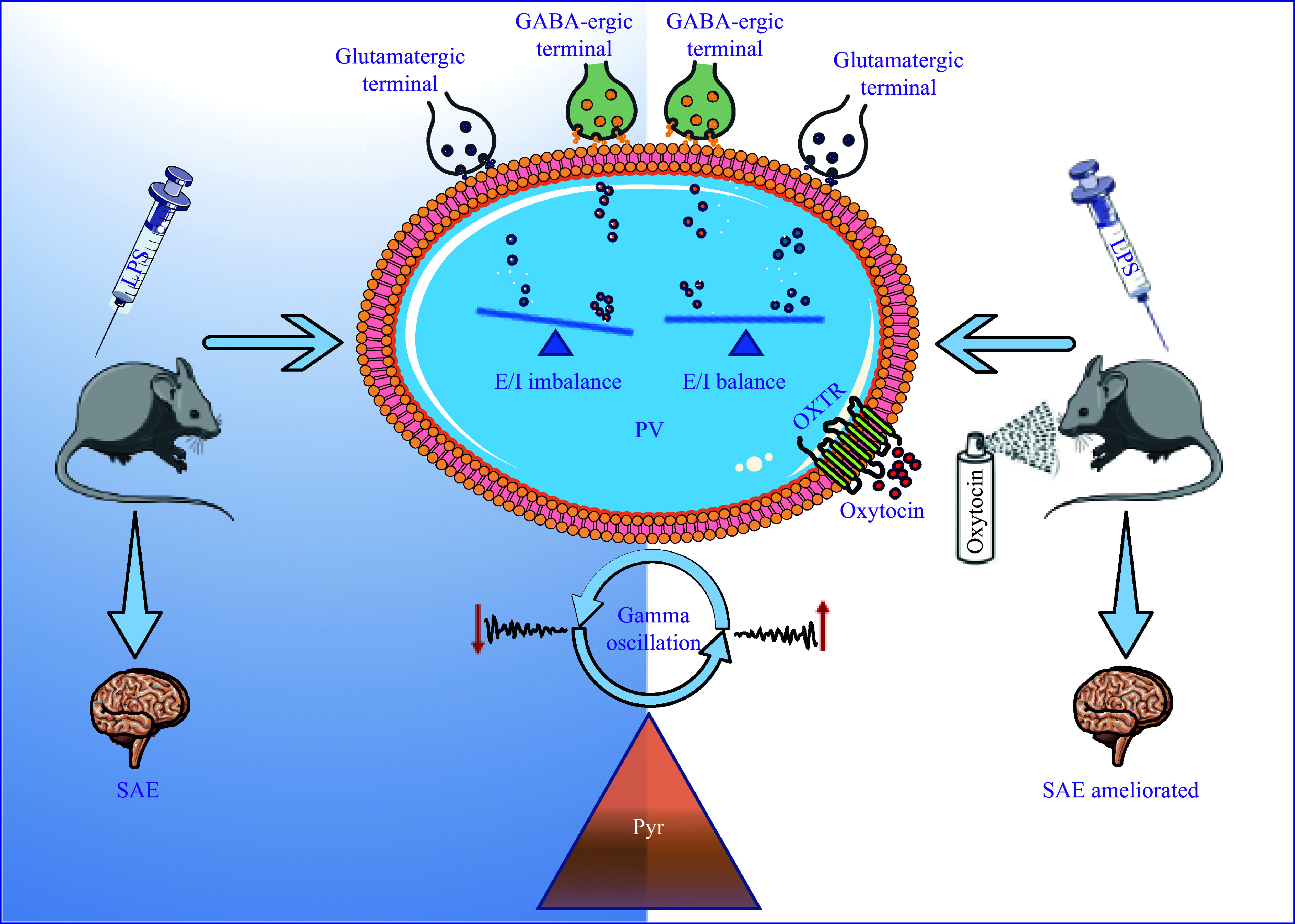
The proposed model of the study. The simplified scheme of oxytocin ameliorates cognitive impairments in the SAE mice. Oxytocin inhalation balances the effects of VGluT1/VGAT on PV^+^ neurons, which subsequently increases the power of gamma oscillations, and the cognitive dysfunctions of the SAE mice are ameliorated. Abbreviations: LPS, lipopolysaccharide; PV, parvalbumin; OA, oxytocin antagonist; GABA, gamma-aminobutyric acid; E/I, excitation/inhibition; Pyr, pyramidal; SAE, sepsis-associated encephalopathy; VGluT1, vesicular glutamate transporter-1; VGAT, vesicular GABA transporter.

In conclusion, the results of the current study indicate that oxytocin administration improves cognitive function in the SAE mice, possibly by stabilizing the excitation/inhibition balance of PV^+^ fast-spiking interneurons and enhancing gamma oscillations (***Fig. 7***). The stabilization of PV interneuron activity and the enhancement of gamma oscillations may play a key role in mitigating behavioral and cognitive impairments in LPS exposure-induced SAE. Together, our findings contribute to a better understanding of behavioral and cognitive impairments in the SAE mice and provide potential targets for exploring effective treatments. However, more specific or comprehensive studies based on advanced methods including optogenetic and pharmacological methods are needed to validate our results.
